# Are there predictive symptoms/signs for a positive oral cow's milk challenge in patients suspected of cow's milk allergy?

**DOI:** 10.1186/1939-4551-8-S1-A31

**Published:** 2015-04-08

**Authors:** Ana Carolina Rozalem, Renata Cocco, Lucila Camargo Oliveira, Marcia Carvalho Mallozi, Dirceu Sole

**Affiliations:** 1 Federal University of Sao Paulo, Brazil

## Background

Cow’s Milk (CM) is the main allergen involved in food allergy in children and it is responsible for the majority of Oral Food Challenges in our unit. The aim of this study was to describe differences between children with negative and positive CM food challenge (CMFC).

## Methods

128 children with suspected CMA were undergone to CMFC (June/2007 to Feb/2014) and comprised two groups according to the result: negative test (passed, PG, n=100) and positive test (failed, FG, n=28). Both groups were analyzed regarding to age at first reaction, gender, nutritional status, breastfeeding, familial history of FA, symptoms reported, presence of asthma, allergic rhinitis or atopic dermatitis and results of skin prick test (SPT). FG was analyzed according to required amount of CM to elicit reaction, symptoms and severity of reaction.

## Results

Comparing both groups, FG was significantly associated with patient’s reports of urticaria (79% x 40%, OR=5.5; 95%IC:2.1-14.8; p=0.05), pruritus (46% x 7%; OR=28.0; 95%IC:7.1-110.1; p<0.0001), vomiting (54% x 26%; OR=3.3; 95%IC:1.4-7.8; p=0.01), rhinoconjuctivitis (18% x 5%; OR=4.1; 95%IC:1.1-15.5; p=0.04) and anaphylaxis (36% x 16%; OR=2.9; 95%IC:1.1-7.5; p=0.03). Gender, age at first reaction, exclusively breasfeeding, familial history of FA, presence of other atopic diseases and diameter of SPT (histamine, CM and milk fractions) did not differ between the groups. Less than 10% of all children were underweight. The median amount of CM to elicit a reaction during CMFC was 13mL and it was not related to severity of symptoms, presence of atopic diseases or age at first reaction. During CMFC, 50% of FG patients presented with urticaria and 14%, an anaphylactic reaction.

## Conclusions

Although CMA has been frequently suspected in Brazilian children, just a little amount (21.8%) really confirmed the diagnosis. There were no specific clinical characteristics for a positive CMFC reaffirming the need of CMFC for the diagnosis of CMA.

